# Multiple Determinations of Sperm DNA Fragmentation Show That Varicocelectomy Is Not Indicated for Infertile Patients with Subclinical Varicocele

**DOI:** 10.1155/2014/181396

**Published:** 2014-05-20

**Authors:** Agustín García-Peiró, Jordi Ribas-Maynou, María Oliver-Bonet, Joaquima Navarro, Miguel A. Checa, Alexandros Nikolaou, María J. Amengual, Carlos Abad, Jordi Benet

**Affiliations:** ^1^Departament de Biologia Cellular, Fisiologia i Immunologia, Facultat de Medicina, Universitat Autònoma de Barcelona, 08193 Bellaterra, Spain; ^2^Càtedra de Recerca Eugin, Universitat Autònoma de Barcelona (UAB), 08193 Bellaterra, Spain; ^3^Centro de Infertilidad Masculina y Análisis de Barcelona (CIMAB), Edifici Eureka, PBM5, Parc de Recerca de la UAB (PRUAB), 08193 Bellaterra, Spain; ^4^Departament de Obstetricia i Ginecologia, Parc de Salut Mar, Universitat Autònoma de Barcelona, 08003 Barcelona, Spain; ^5^Laboratorio de Andrología, CIRH, Clínica Corachan, ANACER, 08017 Barcelona, Spain; ^6^UDIAT, Centre Diagnòstic, Corporació Sanitària Parc Taulí, Institut Universitari Parc Taulí, UAB, 08208 Sabadell, Spain; ^7^Servei d'Urologia, Corporació Sanitària Parc Taulí, Institut Universitari Parc Taulí, UAB, 08208 Sabadell, Spain

## Abstract

Varicocele is one of the most common causes of low semen quality, which is reflected in high percentages of sperm cells with fragmented DNA. While varicocelectomy is usually performed to ameliorate a patient's fertility, its impact on sperm DNA integrity in the case of subclinical varicocele is poorly documented. In this study, multiple DNA fragmentation analyses (TUNEL, SCD, and SCSA) were performed on semen samples from sixty infertile patients with varicocele (15 clinical varicoceles, 19 clinical varicoceles after surgical treatment, 16 subclinical varicoceles, and 10 subclinical varicoceles after surgical treatment). TUNEL, SCD, and SCSA assays all showed substantial sperm DNA fragmentation levels that were comparable between subclinical and clinical varicocele patients. Importantly, varicocelectomy did improve sperm quality in patients with clinical varicocele; however, this was not the case in patients with subclinical varicocele. In summary, although infertile patients with clinical and subclinical varicocele have similar sperm DNA quality, varicocelectomy should only be advised for patients with clinical varicocele.

## 1. Introduction

One of the main causes of male infertility stems from a series of abnormally dilated veins in the pampiniform plexus, commonly called varicocele. Its presence and severity are often associated with impaired spermatogenesis and poor sperm quality [1]. Varicocele incidence has been estimated to be 21%–41% in the infertile male population [2, 3]. Concerning its diagnosis, clinical varicocele is determined according to the Dubin grading system during physical examination, while subclinical varicocele is typically detected by scrotal Doppler ultrasonography [4, 5]. Distinction between clinical and subclinical varicoceles is important, as urologists must choose the most suitable (surgical) method depending on the patient's clinical state to improve fertility [6]. This is not an easy task, particularly in the case of subclinical varicocele. Not only is there a lack of data about subclinical varicocele on sperm parameters, but also surgical treatment of subclinical varicocele is currently debated, as contradictory postoperative results are being reported [7].

As a measure of sperm quality, sperm DNA fragmentation (SDF) has experienced a growing interest in recent years [8]. Sperm DNA damage is now linked to longer conception times [9], higher miscarriage rates [10, 11], and even severe childhood diseases such as cancer or neurological disorders [12]. Main mechanisms of SDF in the sperm cell are nuclease activation in an apoptotic-like process and oxidative stress associated with a defective maturation and nuclear protamination [13, 14]. Typically, high percentages of sperm cells with fragmented DNA are found in varicocele patients [15, 16]. Surgical treatment is reported to improve SDF levels [17, 18]; however there is a lack of information about sperm DNA integrity in subclinical varicocele as well as conflicting results about the impact of vein repair [7].

The objective of this study was therefore to characterize the degree of sperm DNA fragmentation using three different methodological approaches in four cohorts of infertile males: (i) clinical varicocele without varicocelectomy, (ii) clinical varicocele after varicocelectomy, (iii) subclinical varicocele without varicocelectomy, and (iv) subclinical varicocele after varicocelectomy.

## 2. Materials and Methods

### 2.1. Selection of Patients

The study included a total of 60 infertile males with varicocele who were classified in four different cohorts. The first cohort included 15 males with nontreated grade I clinical varicocele (CV), the second group included 16 males with subclinical varicocele diagnosed by scrotal Doppler ultrasonography (ScV), the third cohort included 19 patients with surgically treated clinical varicocele (T-CV), and the last cohort included 10 patients with surgically treated subclinical varicocele (T-ScV). Samples from surgical treated patients were obtained 6 to 12 months after the varicocelectomies were performed (Buntz method). The age of all donors ranged from 25 to 35 years. Patients with genitourinary inflammation, leukocytospermia, or altered hormonal profiles were excluded from the study. Written informed consent was obtained from all patients and the Institutional Ethics Committee approved the study.

### 2.2. Sample Collection

Semen samples were obtained by masturbation after three days of sexual abstinence. Prior to cryopreservation, fresh ejaculate was allowed to liquefy. Then, samples were mixed 1 : 1 with cryopreservation medium (14% glycerol, 30% egg yolk, 1.98% glucose, and 1.72% of sodium citrate), aliquoted and incubated at −80°C in an isopropanol bath overnight, and then plunged into liquid nitrogen until the experiment was performed. For analysis, all samples were thawed by immersion in a 37°C water bath for 30 seconds and washed three times with PBS buffer at room temperature, and the sperm concentration was adjusted according to the requirements for TUNEL [20], SCD [21], and SCSA [22].

### 2.3. Terminal Transferase dUTP Nick-End Labeling (TUNEL) Assay

For the TUNEL assay, the* in situ* cell-death detection kit (Roche Diagnostic GmbH, Penzberg, Germany) was used as previously described [20]. This assay quantifies, by flow cytometry or fluorescent microscopy, the incorporation of labeled deoxyuridine triphosphate (dUTP) at the sites of DNA breaks in a reaction catalyzed by the deoxynucleotidyl transferase enzyme. Semen samples were washed twice in PBS and the concentration was adjusted to 10 × 10^6^ cells/mL. 200 *μ*L of this sperm suspension was fixed in an equal volume of 4% (w/v) paraformaldehyde for 1 hour at room temperature and then washed in PBS supplemented with 1% (v/v) bovine serum albumin (BSA; Sigma Chemicals). Sperm cells were permeabilized using 0.1% (v/v) Triton X-100 in 0.1% (w/v) sodium citrate for 2 min on ice and then washed twice in PBS supplemented with 1% BSA. The pellet was incubated in 50 *μ*L of a mix containing 45 *μ*L of the label solution plus 5 *μ*L of the terminal deoxynucleotidyl transferase (TdT) enzyme for 1 hour at 37°C in the dark. The sample was then washed twice using 1% BSA in PBS. The negative control was incubated without the TdT enzyme and the positive control was prepared before the labeling reaction with an additional treatment with DNase I (Roche Diagnostic GmbH, Penzberg, Germany), 100 IU, for 10 min at 37°C. In order to perform flow cytometry analysis, the final pellet from the sperm sample was resuspended in a final volume of 1 mL PBS. Green fluorescence (TUNEL-positive cells) was measured using a 530 nm ± 30 nm band-pass filter. A total of 10,000 events were measured at a flow rate of 200–300 cells/s on a flow cytometer (FACSCalibur, Becton Dickinson, NJ, USA). Data were processed by CELLQUEST analysis software (Becton Dickinson).

### 2.4. Sperm Chromatin Dispersion Test (SCD)

For the SCD test, the Halosperm kit was used (Chromacell SL, Madrid, Spain). It is based on the principle that sperm with fragmented DNA fails to produce the characteristic halo of dispersed DNA loops that is observed in healthy nonfragmented sperm DNA [21]. The semen samples were washed twice in PBS and the concentration was adjusted to 5 × 10^6^ cells/mL. Low-melting-point agarose was melted in a water bath at 90°C–100°C for 5 min and then placed in water at 37°C for 5 min. 60 *μ*L of the semen sample was then mixed with agarose and 20 *μ*L of the semen-agarose mixture was pipetted onto an agarose-coated slide, covered with a coverslip and left at 4°C for 5 min. The coverslip was gently removed and the slide was immersed in an acid solution for 7 min, washed for 5 min with distilled water, and incubated in 10 mL of the lysing solution for 25 min. After washing, the slides were dehydrated in 70%, 90%, and 100% ethanol for 2 min each and then air-dried. Slides were stained for bright-field microscopy using Diff-Quick (2 *μ*g/mL) (Panreac, Barcelona, Spain) according to manufacturer's instructions. 300 spermatozoa were scored and the proportion of sperm with fragmented DNA was expressed as % SDF.

### 2.5. Sperm Chromatin Structure Assay (SCSA)

SCSA protocol has been described elsewhere [22]. Briefly, an aliquot of the thawed semen sample was diluted to a concentration of 2 × 10^6^ sperm/mL in TNE buffer (0.15 M NaCl, 0.01 M Tris, and 1 mM EDTA, pH 7.4) to a total of 200 *μ*L. Thereafter, 400 *μ*L of acid detergent solution (0.08 M HCl, 0.15 M NaCl, and 0.1% Triton X-100, pH 1.2) was added. After 30 s, sperm cells were stained by adding acridine orange (AO) staining solution (Life Sciences, Oregon, USA), containing 6 *μ*g AO per mL buffer (0.037 M citric acid, 0.126 M Na_2_HPO_4_, 1.1 mM EDTA, and 0.15 M NaCl, pH 6.0). After 3 min staining, a total of 5,000 sperm cells were analyzed by flow cytometry (FACSCalibur, Becton Dickinson, NJ, USA). The percentage of sperm with DNA fragmentation was determined in the main sperm population and detected as increased red fluorescent signal compared to intact sperm DNA fluorescence.

### 2.6. Statistical Analysis

Data analysis was performed using the Statistics Package for the Social Sciences software, version 15 (SPSS Inc., Chicago, IL). SDF values were compared using the Mann-Whitney *U* test. Correlations were studied using the Spearman test. The level of significance was established at 95% of the confidence interval in order to be considered statistically significant.

## 3. Results

### 3.1. Multiple Determination of Sperm DNA Fragmentation

**Figure 1:**
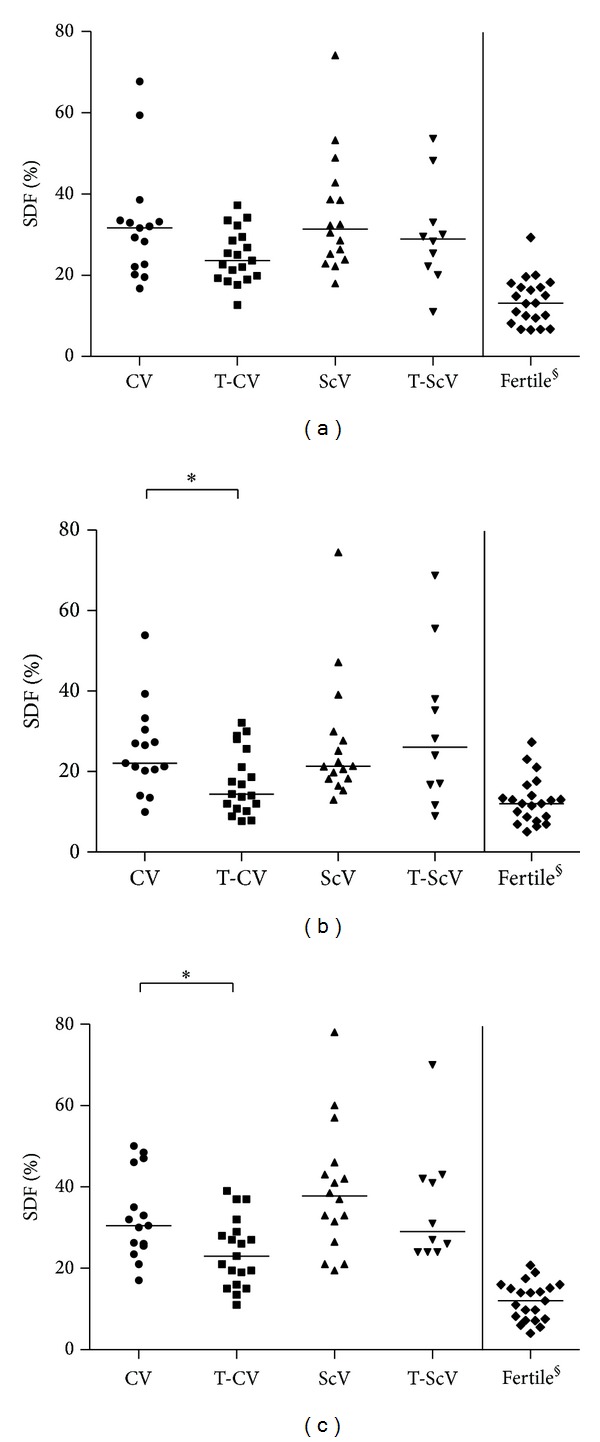
Sperm DNA fragmentation (SDF) values as determined by terminal transferase dUTP nick-end labeling (TUNEL) (a), sperm chromatin structure assay (SCSA) (b), and sperm chromatin dispersion (SCD) (c) assays. CV, nontreated grade I clinical varicocele; T-CV, surgically treated clinical varicocele; ScV, nontreated subclinical varicocele; T-ScV, surgically treated subclinical varicocele. Horizontal bars represent median SDF values. *P < 0.05; ^§^data of fertile donors from [19].

SDF values of each patient as determined by TUNEL, SCSA, and SCD are depicted in Figure 1. Median and range of SDF values obtained for all patient groups are shown in Table 1. Clinical and subclinical varicocele groups without varicocelectomy (i.e., CV versus ScV) displayed substantial sperm DNA damage regardless of the method of analysis used. Patients with clinical varicocele that had undergone varicocelectomy showed significant lower SDF values compared to the untreated group (i.e., T-CV versus CV), in both SCD (*P* < 0.05) and SCSA assays (*P* < 0.05), but failed to reach significance in the TUNEL assay (*P* = 0.09). However, in patients with subclinical varicocele, no benefit of varicocelectomy on SDF values was found by any of the methods (i.e., ScV versus T-ScV).

### 3.2. Correlation Analysis

A good correlation amongst the three methods to determine sperm DNA damage was observed. Values were *r* = 0.703 (*P* < 0.001) for TUNEL versus SCSA, *r* = 0.568 (*P* < 0.001) for TUNEL versus SCD, and *r* = 0.662 (*P* < 0.001) for SCSA versus SCD.

## 4. Discussion

In this work we attempted to answer three questions. (i) Are TUNEL, SCD, and SCSA assays equally suited to determine sperm DNA damage in varicocele patients? (ii) Is sperm DNA quality comparable between infertile patients with clinical varicocele or subclinical varicocele? (iii) Does varicocelectomy improve sperm DNA quality in clinical and subclinical varicocele patients?

Currently, there is a debate about whether the various methods available to determine the degree of sperm DNA fragmentation offer the same guarantees of sensitivity and specificity [12, 19]. One issue is the occurrence of apoptotic bodies which, due to their similar forward scatter/side scatter properties as sperm cells, complicate the interpretation of results in the TUNEL assay [24]. In addition, the presence of these apoptotic bodies is especially common in patients with asthenoteratozoospermia, such as varicocele patients [25]. It is therefore likely that our results obtained by TUNEL were biased by this phenomenon, which is substantiated by the higher correlation rates we observed in a previous study when using fertile subjects with normal seminal parameters [26]. In consequence, apoptotic bodies must be taken into account in cytometric assays, such as TUNEL, particularly in the case of varicocele patients. Regarding the sensitivity of TUNEL, SCD, and SCSA, continuous efforts are being exerted to optimize these methods. More recently, it is even possible to determine whether DNA damage is single-stranded or double-stranded by alkaline and neutral comet assays [27, 28].

Nevertheless, our TUNEL, SCD, and SCSA analyses unequivocally showed that infertile patients with diagnosed subclinical varicocele have similar (poor) sperm DNA quality as infertile patients with clinical varicocele (Figure 1). In addition, significant amounts of highly DNA degraded sperm (DDS) were encountered in both patient groups (Table 1). This sperm subpopulation, which is defined by massive protein depletion and DNA damage, typically constitutes 1%–4% of healthy semen. In contrast, up to eightfold higher DDS levels are characteristic of patients with varicocele, even when compared to other infertile males [23, 27]. These high DDS levels in subclinical and clinical varicocele would imply a similar negative impact for these groups of patients on spermatogenesis and a corresponding difficulty in achieving pregnancy.

The last point of interest is the putative beneficial effect of surgical treatment by the Buntz method varicocelectomy on sperm DNA quality. Operated patients with clinical varicocele displayed significantly lower SDF levels compared to the untreated group (Table 1), which is in line with previous studies reporting increased sperm DNA quality and improved pregnancy rates [29–31]. In contrast, we observed no benefit of varicocelectomy on SDF values in patients with subclinical varicocele. One plausible explanation for this discrepancy is the difficulty for the surgeon to specifically target the affected veins. Despite the fact that both types of varicocele have substantial SDF levels and thus would require the same treatment, results obtained in this work indicate that varicocelectomy of subclinical varicocele does not confer any amelioration. Varicocelectomy by the Buntz method should therefore be counterindicated in patients with subclinical varicocele. A promising alternative is the microsurgical varicocelectomy, performed by a subinguinal or inguinal incision [32]. Recent studies show that better results are obtained by microsurgical varicocelectomy [6] and in particular that sperm DNA quality is ameliorated in patients with clinical varicocele [17, 18, 33]. The next step would therefore be to assess the efficacy of microsurgical varicocelectomy in the case of subclinical varicocele.

In conclusion, using multiple sperm DNA determination methods we showed (i) that clinical and subclinical varicoceles have a similar negative effect on sperm DNA integrity in infertile patients and (ii) that varicocelectomy improves sperm DNA quality in clinical but not in subclinical varicocele patients. Alternative treatments such as microsurgery should thus be explored for subclinical varicocele patients.

## Figures and Tables

**Table 1:** Multiple determination of sperm DNA fragmentation (SDF) in infertile males with nontreated grade I clinical varicocele (CV), surgically treated clinical varicocele (T-CV), nontreated subclinical varicocele (ScV), and surgically treated subclinical varicocele (T-ScV). Highly DNA degraded sperm (DDS) was also measured by the sperm chromatin dispersion (SCD) assay. Median SDF values are given and ranges are bracketed. Pairwise comparisons were performed using the Mann-Whitney *U* test.

Type of SDF assay	CV (*n* = 15)	T-CV (*n* = 19)	ScV (*n* = 16)	T-ScV (*n* = 10)	Fertile (*n* = 21)
TUNEL (%)	31.60 (16.79–67.71)	23.65 (12.68–37.22)	31.38 (17.99–74.10)	28.95 (11.06–53.65)	13.14^§^ (6.59–29.33)
SCSA (%)	22.11 (9.98–53.88)	14.39* (7.70–32.19)	21.30 (12.94–74.48)	26.12 (8.99–68.77)	12.00^§^ (5.00–27.31)
SCD (%)	30.50 (17.00–50.00)	23.00* (11.00–39.00)	37.75 (19.50–78.00)	29.00 (24.00–70.00)	12.00^§^ (4.00–20.80)
DDS (by SCD) (%)	17.00 (5.50–30.00)	13.00 (6.00–22.50)	19.50 (11.50–37.00)	17.25 (10.00–30.00)	1.20^#^ (0.00–4.50)

*P < 0.05.

^§^Data from [19].

^#^Data from [23].
